# Unilateral Discomfort Increases the Use of Contralateral Side during Sit-to-Stand Transfer

**DOI:** 10.1155/2017/4853840

**Published:** 2017-04-26

**Authors:** Simisola O. Oludare, Charlie C. Ma, Alexander S. Aruin

**Affiliations:** ^1^Department of Physical Therapy, University of Illinois at Chicago, Chicago, IL 60612, USA; ^2^PhD Program in Rehabilitation Sciences, College of Applied Health Sciences, University of Illinois at Chicago, Chicago, IL 60612, USA

## Abstract

Individuals with unilateral impairment perform symmetrical movements asymmetrically. Restoring symmetry of movements is an important goal of rehabilitation. The aim of the study was to evaluate the effect of using discomfort-inducing devices on movement symmetry. Fifteen healthy individuals performed the sit-to-stand (STS) maneuver using devices inducing unilateral discomfort under the left sole and left thigh or right sole and right thigh and without them. 3D body kinematics, ground reaction forces, electrical activity of muscles, and the level of perceived discomfort were recorded. The center of mass (COM), center of pressure (COP), and trunk displacements as well as the magnitude and latency of muscle activity of lower limb muscles were calculated during STS and compared to quantify the movement asymmetry. Discomfort on the left and right side of the body (thigh and feet) induced statistically significant displacement of the trunk towards the opposite side. There was statistically significant asymmetry in the activity of the left and right Tibialis Anterior, Medial Gastrocnemius, and Biceps Femoris muscles when discomfort was induced underneath the left side of the body (thigh and feet). The technique was effective in causing asymmetry and promoted the use of the contralateral side. The outcome provides a foundation for future investigations of the role of discomfort-inducing devices in improving symmetry of the STS in individuals with unilateral impairment.

## 1. Introduction

It is known that individuals with a unilateral impairment such as stroke show a characteristic asymmetry of gait, posture, and weight bearing in favor of the nonparetic leg [1–4]. This leads to the learned disuse of the more affected side of the body, a condition where patients learn to use the stronger side of their bodies while neglecting the weaker side [2]. While this compensation may be expedient for some patients, learned disuse can also lead to greater muscle weakness on the affected side resulting in poorer performance of daily activities [5].

The standard approach to minimize the learned disuse of the upper limb is the constraint-induced movement therapy (CIMT) [6]. CIMT is an approach in which a patient's stronger limb is constrained in order to force the patient to use the weaker limb. This approach has been successful in restoring function to the upper limbs of patients with stroke, traumatic brain injury, and other disorders [7–9]. However, CIMT, as it was developed, is restricted to treating the upper limb and no equivalent therapy has been created to target the lower limb [10]. A possible reason for this is that movements generated by the lower limb such as locomotion and sit-to-stand maneuver are bilateral movements which cannot be adequately performed if both limbs are nonfunctional, one due to impairment and the other because of constraint [11]. As a result, constraining the stronger lower limb may not produce the desirable result of forced use in a patient with unilateral movement disorders.

Nevertheless, the success of the CIMT prompted the development of many forms of “forced use” therapies and has made it possible to apply forced use to the lower limbs. One of those approaches is the Compelled Body Weight Shift Therapy (CBWST). CBWST involves the use of a shoe insert that establishes a lift of the nonaffected lower extremity to force the patient to shift their body weight towards the more affected lower extremity [10]. The CBWST approach involving the use of a flat (smooth) lift under the nonparetic leg has been found to improve stance weight bearing symmetry in individuals with stroke [10, 12, 13]. Multisession therapy using the CBWST approach has also been found to be helpful in the restoration of symmetry of stance and in improvement of gait velocity in individuals with acute stroke [14] and chronic stroke [15]. Approaches used to facilitate the utilization of the more impaired lower limb during sit-to-stand involve asymmetric positioning of the lower limbs [16] and the use of blocks below the unaffected feet similarly to CBWST [17]. Thus, it has been shown that both asymmetrical feet placement and blocks are able to increase weight bearing of the more impaired limb in patients with a hemiparetic stroke performing sit-to-stand task.

Another approach to facilitate forced use of a limb is the utilization of nociceptive feedback via induced discomfort [18]. Unilateral discomfort has been shown to cause changes in postural control and movement control in healthy adults and also in patients with neuromuscular deficits during locomotion and quiet standing [19]. However, no studies involving experimentally induced discomfort have been performed during the STS task, an important activity of daily living which many patients have difficulty performing.

In this study, we aimed to determine the feasibility of using a new approach of inducing unilateral discomfort in order to produce forced use of the contralateral side during the performance of the STS task. If unilateral discomfort brings about asymmetry of the performance of the STS in healthy individuals, the approach could potentially be beneficial to individuals with unilateral impairment. Thus, our hypothesis was that, during STS, healthy adults will exhibit movement asymmetry when discomfort is induced unilaterally under their thigh and foot. We also hypothesized that when discomfort is induced on the left side, movement will be greater on the right side and vice versa.

## 2. Methods

### 2.1. Subjects

Fifteen healthy young adults (8 males, 7 females, 26.7 ± 3.9 years old, height 162.8 ± 8.9 cm, and body mass 66.0 ± 13.0 kg) participated in the study. All subjects were right-limb dominant. They all signed a written informed consent approved by the Institutional Review Board.

### 2.2. Protocol

The subjects were required to sit in a chair positioned on a force platform with both of their feet placed on the top of the platform. The chair (66.0 cm high, 58.5 cm wide and 48.3 cm deep) had a nondeformable wooden seat, arm rests, and no back support. Each subject sat in the chair with a knee flexion angle of 90 degrees and an elbow flexion of 90 degrees. Subjects were required to perform sit-to-stand maneuver with arm support and with or without unilateral discomfort. The experimental protocol began with a baseline (no discomfort condition) followed by two randomized conditions: (1) standing up using arm support in the presence of discomfort induced on the left side (foot and thigh) (LC) and (2) standing up using arm support with discomfort induced on the right side (foot and thigh) (RC).

Discomfort was induced by tapered devices beneath both the thigh and foot: on the seat under the thigh (approximately 50% distance from the hip joint to the knee joint) and in a standard sandal provided for each subject. The thigh device was a set of 3 evenly spaced pyramidal metal protrusions (base 30 × 40 mm, top 17 mm, and height 35 mm) with center to center distance of approximately 50 mm. The base of the set was 2 mm high with the total height of the device being 37 mm. The foot device was an insole made of polyvinyl chloride embedded with 32 small 3 mm high pyramidal peaks with center to center distance of approximately 10 mm. The base of the insole was 1 mm high with the total height of the insole being 4 mm [19].

Each sit-to-stand trial consisted of sitting for approximately three seconds, standing up at a self-selected speed, and standing for approximately three seconds. Three trials were performed in each experimental condition. In each condition, subjects were asked to rate the level of their perceived discomfort using a 10 cm linear (with one end (0) marked as “no discomfort at all” and the other end (10 cm) as “worst discomfort ever”) Visual Analogue Scale (VAS) [20].

### 2.3. Data Collection and Processing

Three-dimensional kinematic data was collected using a six-camera VICON 612 system (Oxford Metrics, UK). Retroreflective markers were placed over anatomical landmarks bilaterally according to the Plug-In-Gait (PIG) model (Oxford Metrics), which includes second metatarsal head, calcaneus, lateral malleolus, lateral epicondyle of the femur, a marker on the lateral border of the leg (between the lateral malleolus and femoral epicondyle markers), anterior/posterior superior iliac spines, a marker on the lateral border of the thigh (between the femoral epicondyle and anterior superior iliac spines), second metacarpal, lateral epicondyle of the humerus, acromioclavicular joint, and a marker on the lateral border of the arm (between the humeral epicondyle and the acromioclavicular joint markers). Also, subjects wore head and wrists bands with four and two markers attached on them, respectively. Finally, five additional markers were attached over the following landmarks: 7th cervical vertebra (C7), 10th thoracic vertebra, inferior angle of the right scapula, between the two sternoclavicular joints, and xiphoid process of the sternum bone. A lower and upper limb model which estimated joint centers was created using the Plug-In-Gait (VICON) software. The kinematic data obtained from 15 subjects was then filtered with a low pass 4th-order Butterworth filter with a cutoff frequency of 2 Hz. The center of mass (COM) was computed using a rigid body model constructed with fourteen segments [21]. The trunk movement was characterized as the movement of the C7 marker in the rigid body model.

The ground reaction forces and moments of forces were collected via a force platform (Model OR-5, AMTI, USA); the signals were sampled at 5000 Hz. The data was then filtered with a low pass 4th-order Butterworth filter with a cutoff frequency of 20 Hz. The center of pressure (COP) data was computed using methods described in the literature [21].

Electromyographic (EMG) activity of muscles was recorded from the Tibialis Anterior (TA), Medial Gastrocnemius (MG), Rectus Femoris (RF), and Biceps Femoris (BF) bilaterally. Based upon recommendations reported in previous literature [22], disposable electrodes (Red Dot 3M) were attached to the muscle belly of each muscle after cleaning the skin with alcohol wipes. A ground electrode was attached to the anterior aspect of the leg over the tibial bone. EMG signals were collected from nine subjects. The signals were filtered and amplified (10–500 Hz, gain: 2000) with the EMG system (Myopac, RUN Technologies, USA). The raw signals were filtered with a high pass 2nd-order Butterworth filter with a cutoff frequency of 20 Hz. The signals were then full wave rectified and filtered with a low pass 2nd-order Butterworth filter with a cutoff frequency of 2 Hz. The onset of muscle activity was determined by an algorithm which detected the moment when muscle activity surpassed baseline activity [23]. The amplitude of the muscle activity was computed as the integral of the muscle activity from the movement onset to standing and latency was computed as the difference between the movement onset and muscle activity onset.

The VICON 612 data station controlled data collection of all signals: forces, moments of force, and EMG signals were acquired at 5000 Hz and kinematic data were collected at 100 Hz.

### 2.4. Data Analysis

Center of mass position, trunk movement, center of pressure position, and muscle activity were used to quantify the movement. To determine asymmetry, the maximum displacement of each movement variable to the left and right in the discomfort conditions was computed and compared. Before comparing the maximum displacement, each movement variable was normalized by subtracting the magnitude of the variable during the baseline condition from the magnitude of the same variable in the discomfort conditions. To ensure that the portion of the movement being subtracted was in phase, sitting, stand up, and standing phases of the task were normalized (via interpolation) to 100% of the period before the movement normalization was performed. The determination of the start and end of the sitting, stand up, and standing phases was done using the ground reaction force data [24] and validated by the center of mass velocity [25]. To determine the symmetry of the muscle activity, the activity in the left muscle was compared to the activity in the right muscle. All data analysis was performed using MATLAB R2014 b (MathWorks, MA, USA).

### 2.5. Statistical Analysis

A paired Student's *t*-test was used to determine if the discomfort levels induced in both conditions were statistically significantly different by comparing the VAS scores (*p* = 0.05). A paired Student's *t*-test was used to determine if the maximum displacements of the COP, COM, and trunk to the left or right were significantly different between both conditions (*p* = 0.05). The differences in muscle activity between left and right muscles in each condition were examined using a paired Student's *t*-test (*p* = 0.05). All statistical analyses were performed using SPSS v23 (IBM, NY, USA).

## 3. Results

### 3.1. Discomfort Levels

The level of the perceived discomfort in the baseline (no discomfort) and RC and LC conditions were 0 cm, 1.7 cm, and 1.5 cm, respectively. The discomfort level in each of the RC and LC conditions was statistically different from the baseline condition (*p* < 0.05). There was not statistically significant difference between RC and LC conditions (*p* > 0.05).

### 3.2. Duration of STS Performance

The duration of STS in the baseline condition was 2.94 ± 0.88 sec; the durations of STS in the LC and RC conditions were 1.91 ± 0.55 and 1.53 ± 0.27 sec, respectively. Relative to baseline, the durations of STS in the LC and RC were statistically significant (*p* < 0.05). However, the difference between the LC and RC conditions was not statistically significant.

### 3.3. Center of Mass Displacement

The maximum displacement of the center of mass (COM) to the right relative to the baseline in the LC condition was 0.020 ± 0.005 m. The maximum COM displacement to the right relative to the baseline in the RC condition was 0.011 ± 0.018 m. The difference between the LC and RC was not statistically significant.

The maximum COM displacement to the left relative to the baseline in the LC condition was close to zero. The maximum COM displacement to the left relative to the baseline in the RC condition was 0.004 ± 0.016 m. The difference between the LC and RC was not statistically significant.

### 3.4. Trunk Displacement

**Figure 1:**
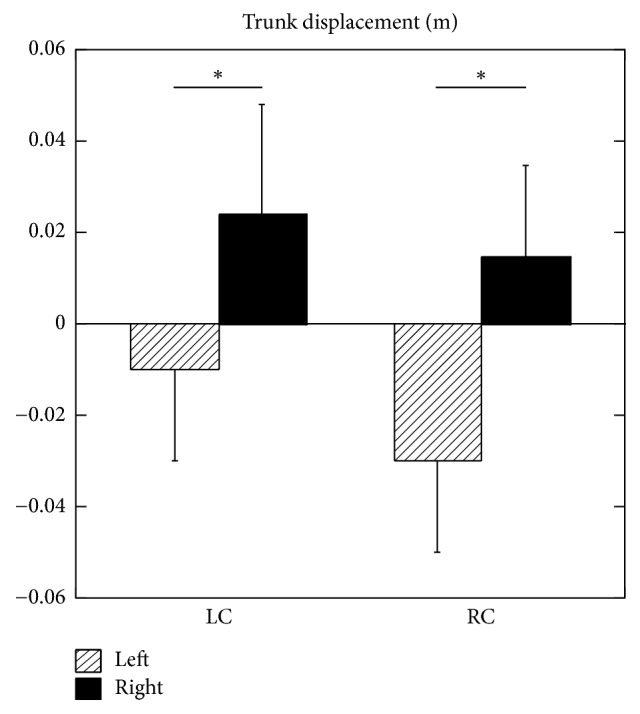
Maximum trunk displacement. LC: discomfort induced on the left side, RC: discomfort induced on the right side. L: left, R: right. *∗* shows statistical significance (*p* < 0.05).

The maximum displacement of the trunk to the right relative to the baseline in the LC condition was 0.024 ± 0.02 m. The maximum displacement of the trunk to the right relative to the baseline in the RC condition was 0.0147 ± 0.02 m. The difference between the LC and RC was statistically significant (*p* < 0.05) (Figure 1).

The maximum displacement of the trunk to the left relative to the baseline in the LC condition was 0.01 ± 0.02 m. The maximum displacement of the trunk to the left relative to the baseline in the RC condition was 0.03 ± 0.02 m. The difference between the LC and RC was statistically significant (*p* < 0.05).

### 3.5. Center of Pressure Displacement

The maximum displacement of the center of pressure (COP) to the right relative to the baseline in the LC condition was 0.06 ± 0.06 m. The maximum COP displacement to the right relative to the baseline in the RC condition was 0.05 ± 0.07 m. The difference between the LC and RC was not statistically significant.

The maximum COP displacement to the left relative to the baseline in the LC condition was 0.03 ± 0.02 m. The maximum COP displacement to the left relative to the baseline in the RC condition was 0.03 ± 0.03 m. The difference between the LC and RC was not statistically significant.

### 3.6. EMG Activity

The latencies of the left and right TA muscles in the LC were 0.47 ± 0.06 s and 0.41 ± 0.04 s, respectively (Table 1). This difference was statistically significant (*p* < 0.05). In the RC conditions the latencies of the left and right TA muscles were 0.39 ± 0.13 s and 0.33 ± 0.09 s, respectively. The difference however was not significant. For the left and right MG muscles the latencies in the LC condition were 0.61 ± 0.15 sec and 0.48 ± 0.11 s, respectively. This difference was statistically significant. The latency of the left and right MG muscles in the RC were 0.37 ± 0.23 s and 0.23 ± 0.28 s, respectively. This difference was not statistically different. The latency of the left BF muscle in the LC was 0.48 ± 0.04 s and it was 0.41 ± 0.04 s, for the right BF muscle. This difference was statistically significant (*p* < 0.05). The latencies of the left and right BF muscles in the RC were 0.38 ± 0.13 s and 0.33 ± 0.08 s, respectively. The difference, however, was not significant. For the left and right RF muscles the latencies in the LC condition were 0.32 ± 0.06 s and 0.50 ± 0.06 s, respectively. This difference was statistically significant. The latency of the left and right RF muscles in the RC was 0.38 ± 0.08 s and 0.46 ± 0.15 s, respectively. This difference was not statistically significant (*p* > 0.05).

**Figure 2:**
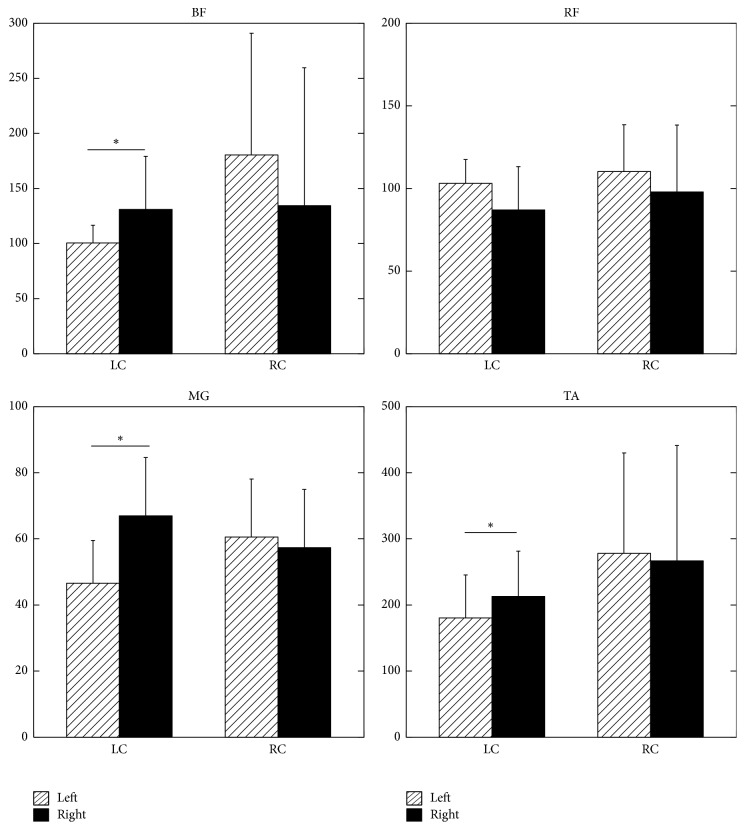
Integrals of EMG activity of the left and right muscles (mV*∗*s). Note increased EMG activity in the muscles on the side contralateral to the side of the induced discomfort. LC: discomfort induced on the left side, RC: discomfort induced on the right side. L: left, R: right. *∗* shows statistical significance (*p* < 0.05).

Integrals of EMG activity of the left and right leg muscles are shown in Figure 2. In general, the activity of a muscle on the side contralateral to the side of the induced discomfort increased indicating asymmetrical pattern. Thus, the integral of the EMG activity of the left Tibialis Anterior (TA) muscle in the LC (a condition with the discomfort induced on the left side) was 180.6 ± 64.9 mV*∗*s and it increased to 213.1 ± 68.4 mV*∗*s in the right TA (*p* < 0.05). The integral of the left TA muscle in the RC was 278.1 ± 151.9 mV*∗*s and it decreased in the right TA to 266.9 ± 174.6 mV*∗*s. However, this difference was not statistically significant. The integral of the left Medial Gastrocnemius (MG) muscle in the LC was 46.66 ± 12.91 mV*∗*s; it was 67.02 ± 17.57 mV*∗*s in the right MG (*p* < 0.05). The integrals of the left and right MG muscles in the RC were 60.55 ± 18.68 mV*∗*s and 57.36 ± 17.57 mV*∗*s, respectively. This difference was not statistically different (*p* > 0.05). The integral of the left Biceps Femoris (BF) in the LC was 100.4 ± 16.1 mV*∗*s and was 130.8 ± 48.2 mV*∗*s in the right BF (*p* < 0.05). The integrals of the left and right BF muscles in the RC were 180.5 ± 110.4 mV*∗*s and 134.3 ± 125.3 mV*∗*s, respectively. This difference was not statistically different (*p* < 0.05). In opposition to the trend, the integrals of the left and right Rectus Femoris (RF) in the LC were 103.1 ± 14.4 mV*∗*s and 86.88 ± 26.3 mV*∗*s, respectively. This difference was not statistically significant. The integrals of the left and right RF muscles in the RC were 110.3 ± 28.14 mV*∗*s and 97.9 ± 40.37 mV*∗*s, respectively. This difference was also not statistically significant.

## 4. Discussion

The aim of this study was to determine whether the device inducing unilateral discomfort increases the use of the contralateral limb in adults performing sit-to-stand task. We hypothesized that, during STS, healthy young adults will exhibit movement asymmetry when discomfort is induced unilaterally under their thigh and foot. The study demonstrated that when experiencing unilateral discomfort, subjects utilized asymmetrical trunk movements and increased the activation of the lower limb muscles on the side opposite to the side of the induced discomfort. Thus, the hypothesis that healthy young adults will exhibit movement asymmetry and thus increased contralateral limb use, when discomfort is induced unilaterally under their thigh and foot, was supported.

Asymmetrical loading during STS is reported in people with unilateral impairment, for example, those who underwent transtibial amputation [26], total knee arthroplasty [27], and anterior cruciate ligament reconstruction [28] and in individuals with stroke [29, 30]. It is described in the literature that when individuals with stroke performed the STS with the paretic foot placed behind the healthy foot, they improved the symmetry of their movement [31]. Moreover, when the unaffected foot of individuals with stroke was placed on a small lift, the EMG activity of muscles in the affected limb recorded during the STS increased and decreased in the unaffected limb [32]. Similar increase in the EMG activity in the muscles on the side opposite to the side of the induced discomfort observed in the current study suggests that the approach indeed could be beneficial to individuals with unilateral impairment.

It is reported in the literature that individuals with a unilateral stroke perform the sit-to-stand task significantly slower than healthy controls [33]. Moreover, it was described that individuals with stroke shortened the rise time after sit-to-stand training in which the feet were positioned asymmetrically (the paretic foot placed posterior) [34]. The subjects in the current study performed STS faster while being exposed to discomfort. Moreover, healthy subjects experiencing discomfort demonstrated changes in the performance of the STS task seen as asymmetrical movements of the trunk as well as the reported asymmetrical pattern of activation of leg muscles. As such, it is tempting to suggest that individuals with unilateral stroke exposed to discomfort on the nonaffected side could perform the sit-to-stand task a bit faster. This suggestion, however, should be tested in experiments involving individuals with stroke.

There are some study limitations. First, the level of discomfort induced in each subject was not customized which resulted in a wide range of discomfort. For this study, performing a regression analysis would not have allowed us to glean a meaningful result. However, future studies should aim to mathematically describe how increasing the level of discomfort affects movement asymmetry and to determine the important variables which control discomfort levels. Secondly and finally, this study focused on healthy young adults and the immediate effect of discomfort on performing the sit-to-stand task.

## 5. Conclusions

When healthy subjects were provided with the discomfort-inducing devices, they performed the sit-to-stand task asymmetrically. The results suggest that if the discomfort is induced on the unaffected side of individuals with unilateral impairment, it can help such individuals to regain the ability to rise from a chair more symmetrically. The outcome of the study provides a foundation for the investigation of the effect of discomfort-inducing devices in rehabilitation of people with unilateral impairments.

## Figures and Tables

**Table 1:** Muscle latency (sec).

Muscle	LC		RC	
	Left	Right	Left	Right
Tibialis Anterior (TA)	0.47 ± 0.06	0.41 ± 0.04^*∗*^	0.39 ± 0.13	0.33 ± 0.09
Medial Gastrocnemius (MG)	0.61 ± 0.15	0.48 ± 0.11^*∗*^	0.37 ± 0.23	0.23 ± 0.28
Biceps Femoris (BF)	0.48 ± 0.04	0.41 ± 0.04^*∗*^	0.38 ± 0.13	0.33 ± 0.08
Rectus Femoris (RF)	0.32 ± 0.06	0.50 ± 0.06^*∗*^	0.38 ± 0.08	0.46 ± 0.15

*∗* shows statistical significance from the left limb during the same condition (LC/RC) (*p* < 0.05).
